# Classification, functions, evolution, and applications of defective viral genomes

**DOI:** 10.3389/fmicb.2026.1921324

**Published:** 2026-08-12

**Authors:** Yunting Xue, Yufang Bi, Chenyang Liu, Zhengjun Yi, Bangyao Sun

**Affiliations:** 1 School of Medical Laboratory, Shandong Second Medical University, Weifang, China; 2 School of Food and Pharmaceutical Sciences, Weifang Vocational College, Weifang, China

**Keywords:** antiviral drugs, defective viral genomes, evolution, vaccine adjuvants, vector

## Abstract

Defective viral genomes (DVGs) are truncated or rearranged products derived from parental viruses during replication and they harbour numerous lethal mutations or large-fragment deletions. Current studies have demonstrated that DVGs play crucial roles in interfering with normal viral replication and activating host innate immunity, suggesting that the presence of DVGs not only may modulate the course of viral infection but also holds substantial potential for applications in antiviral drugs and immune adjuvants. Nevertheless, the mechanisms underlying the functions of different types of DVGs remain incompletely elucidated, thus emphasising the necessity of updating analytical and research methodologies. Consequently, while summarizing the core functions, mechanisms of action, and the evolution of DVGs, this review provides a detailed overview of their classification methods to shed light on the characteristics of DVG formation and functions. In addition, it reviews the current detection technologies of DVGs to enable the identification and accurate quantification of DVGs. Furthermore, the discussion encompasses the distinctive mechanisms of action and the potential applications of DVGs derived from diverse viruses, with the objective of providing novel insights into the development of clinical antiviral drugs.

## 1 Introduction

Defective viral genomes (DVGs) are a class of sub-genomic molecules generated by errors during viral replication. These molecules are distinguished by the partial deletion of genetic components and an inability to complete the replication cycle independently, often manifesting as truncated or structurally distinct forms (Beauclair et al., 2018). As early as the 1940s, researchers observed a class of truncated viral nucleic acid products, incapable of independent replication, in a system of consecutive high-titer multiple passages of influenza virus. They confirmed that these products could significantly and competitively inhibit the proliferation and replication of intact wild-type viruses. These products were termed defective viral genomes (DVGs) (Von Magnus, 1954). In the 1970s, Huang and Baltimore defined defective interference particles (DIPs) as complete particles formed by viral capsid proteins enveloping DVGs (Huang and Baltimore, 1970). Subsequent experiments further confirmed that DVGs themselves lack the genes that encode proteins essential for viral replication. The entire replication, packaging and release process is highly dependent on auxiliary factors, such as RNA polymerase, nucleoproteins and various structural proteins, provided by co-infected cells containing homologous, intact wild-type viruses (Dimmock et al., 1986). DVGs are widely distributed and have been found in both DNA and RNA viruses (Li et al., 2021; Pelz et al., 2023). The mechanism underlying DVG generation remains unclear. The prevailing view held previously was that DVGs are randomly produced due to the lack of proofreading activity of RNA polymerase during high-titer viral replication (Poirier et al., 2016). However, recent studies have indicated that this generation is a consequence of the combined effects of viral protein regulation, RNA recombination, and host RNA editing (Poirier et al., 2016; Sun et al., 2019). For DNA viruses, the generation of DVGs arises from accidental replication termination and erroneous genomic recombination (Addetia et al., 2021), as well as DNA replication dysregulation triggered by aberrant activation of viral origins of replication and interference from host factors(Daniell and Mullenbach, 1978; Tessier et al., 2001), and is rarely driven by the accumulation of single-point mutations(Kaerner et al., 1979).

Conventionally, DVGs have been regarded as byproducts of viral replication *in vitro* unrelated to natural viral infections (Yan et al., 2025). However, studies using various techniques, including high-throughput sequencing, have demonstrated that DVGs exert non-negligible effects on both viral survival and host immunity. On the one hand, they have the capacity to competitively sequester replication resources of standard viruses, such as nucleoproteins and RNA polymerases, thereby directly interfering with viral replication and reducing viral titer and virulence (Fonville et al., 2015). It is imperative to note that, in contrast, specifically structured DVGs have the capacity to function as potent agonists, thereby activating pattern recognition receptors (PRRs) such as retinoic acid-inducible gene (RIG)-1 within the host (Reikine et al., 2014). This, in turn, results in the enhancement of the production of antiviral cytokines to promote host protection (Xu et al., 2015). This demonstrates the promising prospects for developing novel antiviral strategies based on DVGs. Replication-defective viral vectors have achieved successful application in vaccine development, and DIPs with potent interfering activity are also recognized as highly promising next-generation biological antiviral therapeutics (Chaturvedi et al., 2021; Wasik et al., 2018). Meanwhile, it also emphasizes the significance of technological innovations in DVG analytical methods. On this basis, the present review systematically sorts out and briefly summarizes the latest research advances in the field of DVGs. Firstly, a summary of the classification and mechanisms of action of DVGs is presented. Furthermore, given the pivotal role of the isolation and analysis of DVGs in investigating their mechanisms of action during infection, this review also summarises the applications of high-throughput technologies and bioinformatics analyses in DVG research. Finally, we provide an in-depth discussion about the broad application prospects of DVGs in vaccine development and antiviral therapy. A comprehensive understanding of the value of DVGs is therefore required to promote a paradigm shift in virology from “passive response to infections” to “active regulation of infection outcomes.”

## 2 Classification of DVGs

The fundamental attribute of DVGs is attributed to their incomplete genome, which impedes their capacity to complete an autonomous replication cycle. Thereby, all classification methods are centered around this fundamental characteristic, with their logical basis derived from dimensions such as the molecular nature of the defect, the external conditions required for replication, and the specific links of functional defects. The following section will describe two different classification methods.

### 2.1 Classification based on structural characteristics of DVGs

#### 2.1.1 Replication-defective type

**Figure 1:**
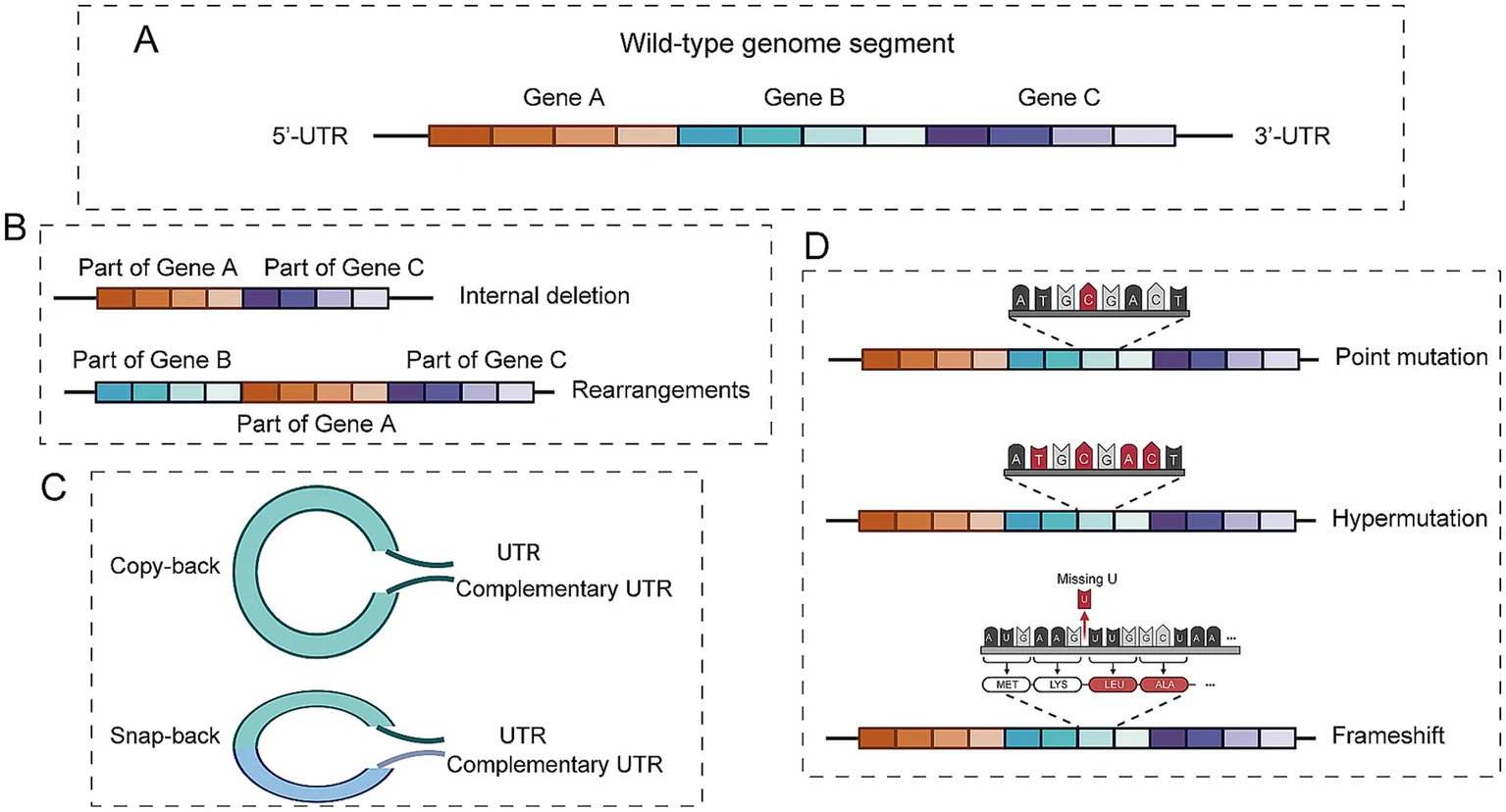
Types of structural variations in DVGs. **(A)** Basic structure of the wild-type viral genome segment. This serves as the reference template for DVG variations. It comprises the 5′-untranslated region (5’-UTR), 3′-untranslated region (3’-UTR), and tandemly arranged Gene A, Gene B, and Gene C. The complete sequence provides the molecular basis for normal viral replication and viral protein expression. **(B)** Structural characteristics of deletion and rearrangement-type DVGs. This category of DVGs belongs to deletion variants, which are truncated genome versions produced when the viral polymerase skips partial genomic sequences during replication. **(C)** Structural characteristics of snap-back and copy-back DVGs. Copy-back DVGs typically arise from the reverse complementary replication of sequences at one end of the genome (most commonly the 5′ end), forming a partially double-stranded panhandle structure. The complementary region covers only a short segment at the genomic terminus, leaving a large single-stranded region in the middle. In contrast, snap-back DVGs are generated by reverse complementary replication across the full length of the genome, resulting in a highly symmetric palindromic structure. **(D)** Mutation-mediated DVG generation forms. This is one of the pathways for DVG formation, including point mutations, hypermutations, and frameshift mutations. These variations can alter viral replication, viral protein expression, and the functional properties of viral proteins.

To date, most studies have suggested that the fundamental nature of DVG generation is attributable to replication defects. However, different types of DVGs also exhibit distinct mechanisms underlying their replication defects compared with the wild-type viral genome (Figure 1A): (i) Deletion DVGs (Figure 1B). Such events generally emerge from substantial internal deletions within the genome, leading to the loss of one or more essential genes (e.g., polymerase genes), while retaining the 5′ and 3′ termini, as well as promoter sequences and cis-acting elements required for replication and packaging (Li and Aaskov, 2014). Due to the loss of a stable intramolecular double-strand initiation element, the RNA-dependent RNA polymerase (RdRp) exhibits very low initiation efficiency and a slow extension rate. During brief extension, a temporary intermolecular double-stranded RNA (dsRNA) forms between the template and the nascent strand, but this dissociates instantaneously upon replication completion (Hoenen et al., 2026). The remaining unpaired bases at the terminus prevent the C-terminal domain (CTD) from tightly binding to the double-stranded terminus, thereby reducing its affinity for pattern recognition receptors (such as RIG-1). The specific generation process of deletion DVGs involves the RdRp pausing during replication due to damaged sites or secondary structures on the template strand. Subsequently, RdRp dissociates from the original template strand and binds to the same or another template strand without connecting to the already synthesized nascent daughter strand, restarting RNA synthesis to form internally deleted daughter strands (Perrault and Semler, 1979). Deletion DVGs have been observed to be produced in abundance during the replication of a variety of positive-sense RNA viruses, including alphaviruses, flaviviruses, and influenza viruses. (ii) Copy-back DVGs (cbDVGs) (Figure 1C). These elements are predominantly identified within negative-sense RNA viruses. Despite the specific generation mechanism remains unclear, the predominant perspective suggests that when the viral polymerase dissociates from the template and rebinds to the nascent strand, binding to a site upstream of the current synthesis site leads to reverse replication, resulting in cbDVGs containing repetitive sequences (Felt et al., 2022; Pye et al., 2025). Sun et al. used the VODKA algorithm to detect breakage/recombination hotspots in respiratory syncytial virus (RSV) and found that breakpoints are distributed in four regions in the upstream and middle parts of the gene, while recombination hotspots are consistently anchored in the 3′ terminal hotspot region, indicating that recombination hotspots are the dominant factor in cbDVG generation (Sun et al., 2019). A distinct form of cbDVGs, designated as snapback DVGs (sbDVGs), is characterized by reverse sequences that extend to nearly the entire genome, comprising a single non-complementary nucleotide and exhibiting no coding capacity. This high degree of complementarity enables them to compete more effectively for the viral replication machinery. Compared with deletion DVGs, the structure of cbDVGs, with complementary sequences at both ends spontaneously forms multiple stable double strands after RNA folding. The flat-end configuration also significantly enhances its affinity for RIG-1. Furthermore, cbDVGs have a far higher affinity for the RdRp template-binding pocket than del-DVGs do, and the truncated sequence further promotes the RdRp synthesis and turnover efficiency (Brennan et al., 2026). (iii) Point mutation, hypermutation, and frameshift mutation DVGs (Figure 1D). Contrary to the findings of preceding studies, recent research has indicated that specific forms of DVGs are attributable exclusively to minor variations. Due to the compact and functionally dense genomes of RNA viruses, point mutations alone can result in deleterious changes. For instance, in studies related to vesicular stomatitis virus (VSV), random alterations or introductions of nucleotide sites revealed that random nucleotide changes are highly likely to be lethal or reduce fitness (Sanjuán et al., 2004). In addition, during adenovirus infection, the cytidine deaminase APOBEC3B accumulates in adenovirus replication centers, triggering C-to-T hypermutation, which also leads to viral genome instability and interferes with the normal expression of its genes. Although such deleterious mutant genomes have not been classified as DVGs, they all interfere with the normal replication of wild-type viruses, forcing them to utilize the missing functions provided by co-infecting wild-type full-length viruses for survival (Vignuzzi and López, 2019).

#### 2.1.2 Packaging-defective type

In comparison with replication-defective DVGs, the distinguishing feature of packaging-defective DVGs is an inherent replication capacity within their genome, yet the inability to assemble into infectious viral particles. The fundamental mechanism underpinning the formation of such DVGs is that, during viral replication, genomic rearrangement or deletion occurs precisely in regions encoding capsid proteins or containing packaging signals (Alnaji et al., 2021; McInerney et al., 2000). Genomic point mutations have been shown to impede the formation of mature viral particles in several viruses. For instance, mutations in the packaging signals of influenza viruses (at the 5′ ends of viral RNA segments 2 and 3) lead to impaired coordination between different viral ribonucleoprotein (vRNP), thus preventing vRNA integration and ultimately resulting in the production of empty particles (Girard et al., 2023). Furthermore, mutations in accessory proteins involved in the regulation of packaging can also cause packaging defects by blocking the recognition and binding between the genome and capsid. This can lead to the accumulation of empty capsids and unpackaged genomes. In the generation of human immunodeficiency virus (HIV)-related DVGs, mutations in the accessory protein Rev. abrogate its binding to the packaging signal (*ψ* sequence) of vRNA. Consequently, the viral genome is prevented from being transported to capsid assembly sites in the cytoplasm, leading to the production of empty capsids (Cockrell et al., 2011).

It has been demonstrated that, since these packaging-defective DVGs are unable to replicate or spread independently (Yamagata et al., 2019), they have the capacity to deliver therapeutic genes into host cells when co-cultured with helper viruses or helper cell lines that harbour packaging genes. This process does not pose a risk of “vector conversion into pathogenic viruses,” and future research focusing on biosafety quantification and process optimization will fully exploit its unique translational merits, thus highlighting their significant application potential (Table 1).

**Table 1:** The comparison of DVG analysis methods.

Analytical methods	Category	Read length	Ability to recognise the structures of DVGs	Scope of application	References
PCR	Standard PCR	/	Can only detect DI with specific breakpoints	Absolute quantification and temporal monitoring of specific DI-RNAs	Gias et al. (2008)
	qPCR				
	RT-PCR				
Nucleic acid sequencing	Illumina short-read sequencing	150–300 bp	Can only detect a single large-scale deletion	Large-scale screening of DI-RNA, rough quantification, and transcriptome-level association analysis	Routh and Johnson (2014)
	PacBio SMRT Sequencing	10–25 kb	Complete identification of single and multiple internal deletions	Analysis of DI-RNA fine structure, investigation of breakpoint characteristics, identification of multiple deletions, and precise quantification of clinical samples	Lui et al. (2019)
	Nanopore sequencing	Tens of kb- Mb	Single deletions can be identified, but multiple deletions in short segments are prone to misinterpretation	FLVGs sequencing, identification of ultra-long DI-RNA, rapid on-site testing	Pye et al. (2025)
	Sanger sequencing	800–1,000 bp	Verification of single-missing fragments in known sequences only	Sequence validation and breakpoint confirmation for a single known DI-RNA	Campagna et al. (2024)
Nucleic acid hybridisation	Northern blot	/	It is difficult to pinpoint the location based solely on a rough distinction in size.	Preliminary screening for the presence of DI-RNA and a rough assessment of its size distribution	Girgis et al. (2022)

### 2.2 Classification by function of DVGs

#### 2.2.1 Defective interfering type

Defective interfering (DI) DVGs are the most functionally well-characterized class among DVGs. Due to their shorter genomes, they possess a replicative advantage, can interfere with the replication of full-length viral genomes, and can be packaged into new viral particles to form DIPs. Specifically, the shorter genome of DIPs enables a faster replication cycle, sequestering replication resources from full-length genomes and leading to a reduction in the yield of infectious progeny viruses. Some DI DVGs, by retaining intact packaging signals, can also compete with full-length viral genomes for viral structural proteins. This results in the occupation of space and resources within viral particles, which in turn reduces the titer of infectious viruses (Kuo and Masters, 2013). Thus, the presence of DIPs also influences the course of viral infection. In various viral infections, DIPs have been shown to attenuate viral pathogenicity, delay infection progression, and even drive infections into a latent or chronic state (Aquino et al., 2025).

#### 2.2.2 Non-interfering type

Non-interfering DVGs, while also defective, exhibit an extremely weak ability to interfere with the replication of full-length viral genomes. As the end products of viral replication, they are unable to autonomously amplify within cells and are instead diluted or eliminated during cell division or death. Bosma et al. emphasized that a significant proportion of DVGs identified in high-throughput sequencing data cannot be confirmed to retain any functional elements due to extensive deletions, and these fragments often lack intact termini or contain non-functional inverted repeats (Bosma et al., 2019). Consequently, non-interfering DVGs cannot even be efficiently recognized and replicated by the viral replicase, exerting little effect on the course of viral infection. Furthermore, it has been observed that some subgenomic particles are capable of harbouring non-functional RNAs. A notable example of this phenomenon is the subviral particles (SVPs) produced by the hepatitis B virus (HBV) (Chai et al., 2008; Rizzetto et al., 1980). Although not strictly classified as DVGs, they are analogous to non-interfering DVGs as defective nucleic acid products lacking complete functional elements. During viral replication, such viral RNAs may, in theory, exert a certain influence on the production of intact viral particles as a result of the consumption of viral structural proteins. However, in reality, they do not possess significant replication-interfering activity and are usually regarded merely as immune decoys to sequester neutralizing antibodies (Hu and Liu, 2017).

## 3 The core function and mechanism of action of DVGs

**Figure 2:**
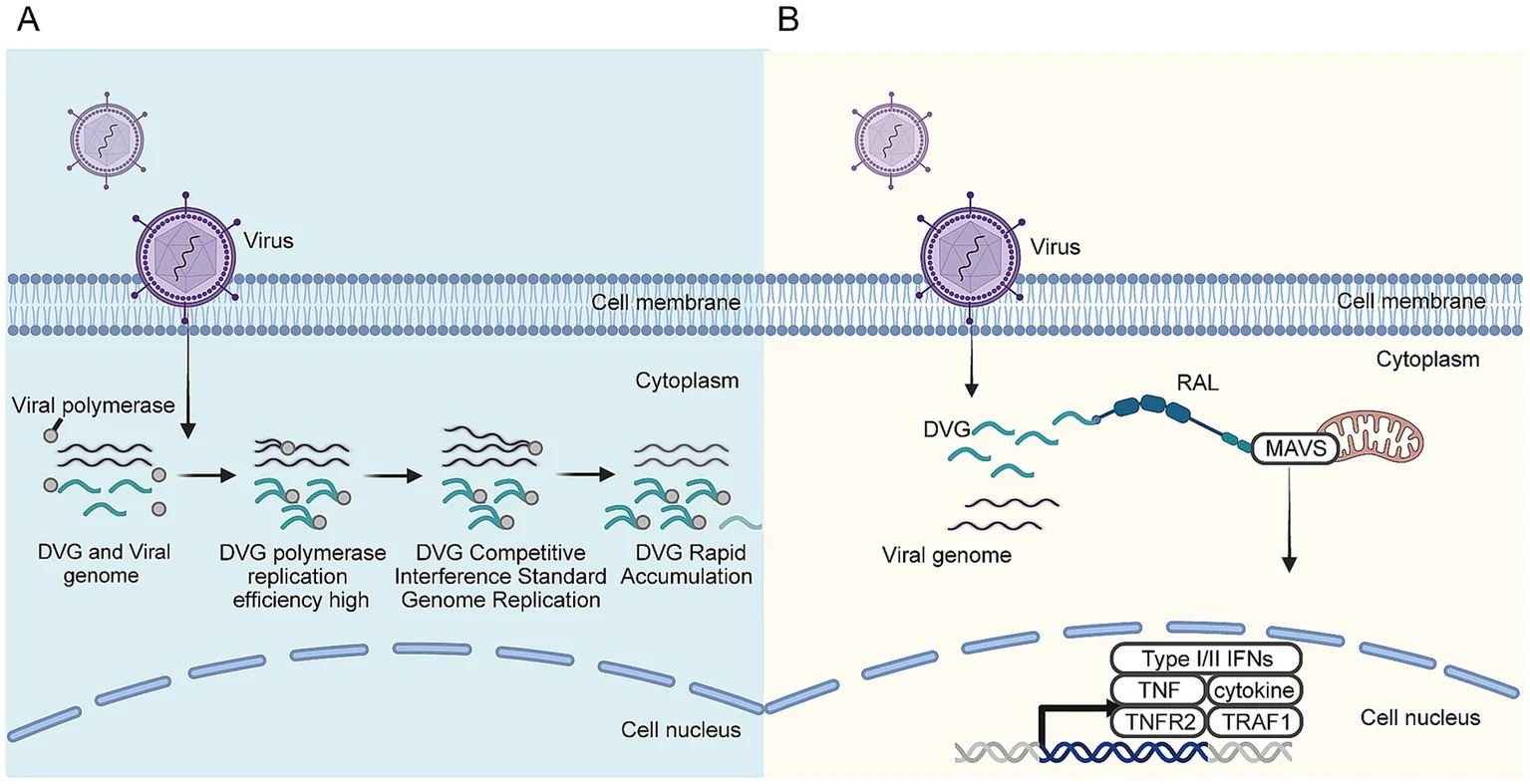
Core mechanism of action of DVGs. **(A)** The process by which DVG interferes with the replication of standard viruses. After a virus infects a host cell, DVG and the wild-type viral genome initiate replication simultaneously. Due to the higher replication efficiency of DVG’s polymerase, it competitively occupies replication resources, interferes with the normal replication of the wild-type viral genome, and ultimately achieves the rapid accumulation of DVG within the cell, thereby inhibiting the proliferation of standard viruses. **(B)** The signaling pathway of innate immune response induced by DVG. DVGs entering the cell can bind to RLR, thereby recruiting and activating MAVS signaling molecules. Through the transmission of this signaling axis, the expression of innate immune effector molecules such as IFNs, TNF, and other cytokines is ultimately induced. Simultaneously, immune regulatory molecules such as TNFR2 and TRAF1 are involved, initiating the host’s innate immune defense.

Despite the absence of complete replication capabilities, DVGs maintain the capacity to disrupt the viral life cycle, modulate the host immune response, and influence viral populations (Figure 2).

### 3.1 Interfering with the viral life cycle

Previous studies have shown that DIP and DVG accumulation in host cells is significantly more efficient than that of full-length viral genomes (Li and Pattnaik, 1997), and it was observed that DVGs with the largest deletions and shortest lengths exhibited the highest replication efficiency. Mechanistically, DIPs and DVGs sequester replication resources from parental helper viruses by virtue of their unique sequence advantages (Liang et al., 2023). The sequence advantages of DVGs are primarily attributable to two scenarios. Firstly, DVGs, particularly cbDVGs, possess efficient flanking trailer promoters that can initiate their own replication more efficiently, preferentially occupying polymerases and proteins required for viral replication (Felt et al., 2022; Re and Kingsbury, 1986). Furthermore, sequence mutations in DVGs have also been found to enhance replication efficiency. A typical example is the DIPs of varicella zoster virus (VZV). Mutations in the promoter sequences of these DIPs have been shown to enhance binding to polymerases and enzymatic proteins, thereby augmenting their replication rate (Rao and Huang, 1982). Duhaut et al. constructed an influenza A virus (IAV) harbouring only a single deleted RNA to investigate the impact of DVGs on packaging. They found that the accumulation of parental segments in infected cells showed no significant difference compared to viruses without deleted RNA, while only the packaging efficiency of parental segments was affected (Duhaut and Mccauley, 1996). This finding indicates that, in addition to sequestering replication resources, some DVGs can preferentially bind to packaging-related proteins, thereby directly reducing the number of infectious helper viruses. In addition, certain perspectives propose that, despite the slower replication rate of longer DVGs in comparison to the shortest ones, the production of mutant proteins may also directly interfere with the generation of intact viruses. For instance, studies have shown that the severe acute respiratory syndrome coronavirus 2 (SARS-CoV-2) expresses the attenuated protein nsp1-10 and these proteins can inhibit helper viruses. However, the specific mechanism remains unclear (Afsar et al., 2022; Banerjee et al., 2020), and further targeted research is required to elucidate the molecular pathways, thereby providing a theoretical basis for optimising the antiviral efficacy of DVGs.

### 3.2 Activate the immune system

DVGs have been shown to exert a significant and unique activating effect on the human immune system. In comparison to wild-type viruses, DVGs are more potent innate immune inducers. They can efficiently trigger the production of type I and type III interferons, thereby initiating a cascade of proinflammatory cytokines (e.g., interleukin (IL)-6, tumor necrosis factor (TNF), IL-1*β*). Furthermore, DVGs enhance adaptive immunity by promoting antigen presentation and dendritic cell maturation. This immune activation strategy contrasts starkly with that of wild-type viruses. RSV infection serves as a pertinent example. The wild-type RSV employs its NS1 and NS2 proteins to inhibit host innate immunity, thereby facilitating high-titer replication and pathogenicity (Ren et al., 2011; Sedeyn et al., 2019). However, studies have confirmed that, when present during infection, DVGs trigger a robust antiviral response in human lung tissue, the intensity of which is positively correlated with the accumulation rate of DVGs (Sun et al., 2015). Notably, the immune response induced by DVGs is concentration-dependent. High levels of DVG accumulation elicit a strong immune response, whereas low DVG concentrations or infection with only wild-type viruses result in minimal immune activation. This leads to spatial heterogeneity in the distribution of immune activation within infected tissues (Genoyer and López, 2019). Immunofluorescence assays have confirmed that DVG-high cells following Sendai virus (SeV) infection are concentrated at the periphery of infection foci, thereby forming a distinct boundary with surrounding uninfected cells. Furthermore, the local interferon (IFN-*β*) concentration is found to be 5–10 times higher than that in other regions. Nevertheless, this concentration dependence is a result of the combination of specific viruses, hosts, and infection sites. The immune interference mechanisms of some viruses may offset the differential effects of local cbDVGs. For instance, measles virus relies on its encoded V and C proteins. The interference effect is enhanced in regions with high cbDVG concentrations and weakened in regions with low concentrations, ultimately homogenizing the global immune response in the brain (Taye et al., 2024).

The fundamental mechanism underlying the immune response-inducing effect of DVGs is attributable to the double-stranded RNA (dsRNA) structures that are generated during viral replication. These structures have been found to retain critical molecular motifs that are recognizable by PRRs of immune cells, such as the 5′ triphosphate group. This has been shown to enhance T cell- and antibody-mediated adaptive immunity. Among the various PRRs, RIG-I within the RIG-like receptor (RLR) family plays the most pivotal role. Indeed, mouse embryonic fibroblasts (MEFs) with a RIG-I gene knockout exhibit reduced IFN-β expression (Mercado-López et al., 2013a). In addition to RIG-I, melanoma differentiation-associated protein 5 (MDA5) can be activated by longer dsRNA molecules, further amplifying immune signals (Killip et al., 2013). However, the efficacy of MDA5 is restricted to a select number of viruses, including the SeV. For other viruses, such as the recombinant measles virus, their defective interfering RNAs (DI-RNAs) feature a structure consisting of a short dsRNA stem and a single-stranded RNA (ssRNA) loop. The ssRNA loop disrupts the continuous long dsRNA structure, thus failing to meet the recognition requirements of MDA5. Consequently, Mura et al. demonstrated that these DI-RNAs showed significant enrichment in RIG-I complexes but no enrichment in MDA5 (Mura et al., 2017).

In addition, a well-characterized property of DVGs is their association with persistent viral infections (Courtney et al., 2026). For instance, cbDVGs have been detected in the testicular tissues of non-human primates infected with Ebola virus. As an immune-privileged site, the testis is regarded as a key location for long-term viral persistence, leading to the speculation that the presence of cbDVGs may be associated with latent viral infections (Diallo et al., 2016). Johnson et al. have demonstrated that the accumulation of Ebola virus DVGs may promote persistent infections through two mechanisms. Firstly, they compete with full-length viruses, inducing periodic fluctuations in the viral replication cycle and thereby reducing the intensity of host immune responses. Secondly, their immune-stimulatory capacity prevents excessive clearance of infected cells, indirectly sustaining viral persistence (Johnson et al., 2021). This mechanism has also been validated in subacute sclerosing panencephalitis (SSPE) caused by measles virus (Taye et al., 2024). Other studies have indicated that there is a correlation between persistent infections by paramyxoviruses and DVGs. When cells are infected with viruses containing high concentrations of DVGs, they can be passaged for at least 17 days, with infectious viruses consistently detected in the supernatant. Conversely, in the absence of DVGs, viral infections result in the demise of all cells within eight days, thereby hindering the establishment of persistent infections (Xu et al., 2017). This process occurs because cells with high DVG concentrations activate the TNFR2/TRAF1 pathway under the regulation of MAVS, which antagonizes TNFα-mediated apoptosis of infected cells (Xu et al., 2017). Research conducted on both SeV and RSV have indicated that cells with elevated concentrations of DVGs from both viruses can survive via the aforementioned pathway and establish persistent infections. Research on the functions of DVG suggests that they may serve as key molecular tools for viruses to regulate the infection cycle and achieve long-term survival (Vignuzzi and López, 2019), highlighting the need to re-evaluate the functions of DVGs in other viruses.

## 4 Methods for the isolation and analysis of DVGs

The isolation and analysis of DVGs is of crucial importance in understanding the viral life cycle. The analysis of DVGs at varying stages of passage or infection provides a means to elucidate their generation mechanisms. For instance, investigating the concentrated regions of truncation sites in influenza virus DVGs can identify high-frequency regions of replication errors (Alnaji et al., 2021; Wu et al., 2022). Furthermore, the identification of isolated DVGs enables the classification of multiple DVG subtypes within a single infection sample (Alnaji et al., 2019; Wang et al., 2020). The temporal fluctuations in these subtypes can be indicative of the virus’s evolutionary trajectory within the host (Martin et al., 2024). Furthermore, DVG production has been observed *in vivo* following the administration of certain live attenuated vaccines, such as the measles vaccine, suggesting that the isolation and analysis of DVGs can be used to evaluate vaccine stability and safety (Calain and Roux, 1988; Pfaller et al., 2015). The following section details the current methods for the isolation and analysis of DVGs.

### 4.1 Density gradient centrifugation

Density gradient centrifugation has been used for the fractional purification of DVGs based on the density differences between DVG-containing viral particles, intact viral particles, and host impurities. It is considered to be one of the primary technologies for the production of high-purity DVG particles at present. The earliest application of density gradient centrifugation to isolate DVGs is documented in the research on Simian Virus 40 (SV40), in which high-passage SV40 virus suspension was subjected to a 48-h centrifugation process in cesium chloride (CsCl). The isolated low-density particles were verified via electron microscopy observation, genomic analysis, and functional validation to exhibit typical DIP characteristics (Yoshiike, 1968). Other gradient media, including sucrose, have also been used for DIP isolation. In the isolation of viral hemorrhagic septicemia virus (VHSV), plaque-purified viruses were further subjected to sucrose density gradient centrifugation. The obtained viral particles were analyzed by SDS-PAGE and fluorescence observation after infecting the *Epithelioma papulosum cyprini* (EPC) cells, confirming that the resulting viral products had ideal purity and infectivity (Rouxel et al., 2016). In comparison with sucrose density gradient centrifugation, the use of iodixanol as a gradient medium has been shown to result in a substantial enhancement of the retention of viral particle activity. Sun et al. established an iodixanol gradient purification protocol for RSV DVGs, with purity verified by qPCR and electron microscopy, where the ratio of DVGs to intact viruses could reach more than 8:1 (Sun and López, 2016). Recently, Harris et al. used iodixanol for the production and purification of recombinant adeno-associated virus (rAAV). This method was found to enhance total viral yield, mitigate the risk of viral precipitation, and further concentrate the final viral product (Harris et al., 2024). However, conventional methods are often challenging when it comes to efficiently capturing, concentrating, and initially purifying viruses from large and complex initial samples. As a centrifugation technique suitable for processing large-volume samples, continuous flow zone centrifugation establishes a continuous-flow rotor with a density gradient. During sample injection, viral particles are selectively captured in specific density zones based on their sedimentation rates. Upon cessation of injection, the separated zones are then sequentially eluted from the rotor and collected in a targeted manner. This method exhibits significant advantages in enabling the large-scale production of DVGs (Cline et al., 1967).

### 4.2 Targeted isolation based on PCR

Polymerase chain reaction (PCR), a widely employed molecular biology technique, has the capacity to specifically amplify the specific sequences contained in DVGs. Among the various PCR-based methods, quantitative PCR (qPCR) and reverse transcription PCR (RT-PCR) are particularly widely used in the isolation and identification of DVGs. qPCR is mainly employed to assess the relative content of DVGs (Table 1). For instance, the LP-BM5 murine leukemia virus model system consists of a replication-defective component (BM5d) and a helper virus (BM5e). A study successfully detected the nucleic acids of both viruses via qPCR two weeks post-infection in mice. This finding indicates this method not only enables quantification but also has certain diagnostic value for the early identification of DVGs (Paun et al., 2005).

In contradistinction to density gradient centrifugation, the function of RT-PCR is not to separate viral particles from impurities, but rather to enhance the purity of target DVG fragments. As a subsequent purification step following density gradient centrifugation, it can avoid interference from residual primers, enzymes, and other contaminants in subsequent ligation and cloning reactions (Gias et al., 2008). The key to the success of this method lies in appropriate primer design strategies. The success of this method is contingent upon the implementation of appropriate primer design strategies. Primers are typically designed to target the non-coding regions of the virus, thereby avoiding the potential for missed detection caused by deletions in the coding regions. Additionally, it is possible to design two rounds of PCR amplification. For instance, universal primers can be used in the first round, while DVG-specific primers can be used in the second. This approach has been shown to improve detection sensitivity. In order to isolate IAV multi-segmented DVGs, Anisi et al. designed primers that bind to the 5′ or 3′ terminal conserved regions of each genomic segment, as recombination of these DVGs only occurs intragenically while the flanking conserved sequences remain intact (Anisi et al., 2025). Sequencing verification confirmed that the products purified by RT-PCR were DVGs containing segmental recombination or deletions (Alnaji et al., 2021; Smith et al., 2021).

### 4.3 Nucleic acid sequencing

The advent of nucleic acid sequencing technology, especially next-generation sequencing (NGS) and third-generation sequencing, including single-molecule real-time fluorescence sequencing (SMRT) and nanopore sequencing, has rendered the large-scale characterization of the diversity of viral DVGs possible (Table 1). The NGS technology facilitates the identification of DVGs through a comprehensive analysis of all genomic fragments present within a sample. A range of bioinformatics tools, including ViReMa (Routh and Johnson, 2014), DItector (Taylor et al., 2025), DVG-profiler (Bosma et al., 2019), and VODKA (Sun et al., 2019), have been developed to facilitate the identification of Illumina short reads containing DVG deletion breakpoints, which are challenging to detect using conventional algorithms. However, it should be noted that there are two fundamental limitations with these tools. Firstly, insufficient optimization for the detection of cbDVGs has been identified as a primary cause of erroneous species identification of cbDVGs. Secondly, the efficiency of NGS data processing is often problematic, resulting in extended analysis time. To address these issues, Sotcheff et al. reported improvements to ViReMa in terms of error handling, data output standardization, and module diversification. Multi-scenario validation with viruses such as HIV and SeV demonstrated that the enhanced ViReMa can accurately capture genetic structural variations (Sotcheff et al., 2023). Achouri et al. developed DG-seq for the detection and quantification of influenza virus DVGs. The validation process was undertaken using *in vitro* transcribed viroid RNA mixtures containing pseudo-DVGs of varying sizes (424–1,184 nt) and frequencies (0.001–0.5). The results showed that DG-seq can stably detect DVGs with a frequency ≥ 10^−2^. Furthermore, using the BWA MEM fast alignment algorithm results in a significant reduction of alignment time for a single sample, ranging from 40 to 90 s (Boussier et al., 2020). Moreover, building on VODKA, which only screens the last 3,000 bp of the reference genome, a new tool, VODKA2, has been developed to encompass the entire genome. This enhancement ensures the comprehensive detection of genetic variations, thereby circumventing the potential for missed detections that may arise from constrained screening regions. Concurrently, it enables parallel analysis of multiple samples via the LSF scheduler, enabling simultaneous processing of bulk RNA-seq data, which is more suitable for large-scale experimental projects (Achouri et al., 2024).

Compared to the short-read sequencing methods, nanopore long-read sequencing can cover the entire cbDVGs or even the full-length viral genomes (FLVGs), demonstrating advantages in identifying large-scale defects and specific single-gene mutations carried simultaneously on the same molecule, such as in defective HIV progenitor viruses and polyomaviruses (Czech-Sioli et al., 2020; Barton et al., 2023). Pye et al. also demonstrated for the first time the existence of distally fragmented cbDVGs in Sendai virus, providing key evidence for the cbDVGs evolution and early infection intermediate state hypotheses (Pye et al., 2025). Another long-read sequencing method, SMRT, which is suitable for tasks requiring high-precision sequencing of medium to long reads. Lui et al. applied SMRT to the targeted detection of DI-RNA in the polymerase gene of the H7N9 avian influenza virus. They fully resolve the full-length defective genomes and also achieved greater sequencing depth, enabling precise quantification of the abundance of various types of DI-RNA (Lui et al., 2019). To investigate the relationship between viral defects within individual H1N1 virus-infected cells and immune activation, Russell et al. additionally incorporated single-cell sequencing to correlate viral defects with IFN quantification in individual cells (Russell et al., 2019). They found that several types of viral defects, including NS deletions, PB1 mutations and large PB1 deletions, significantly enhanced IFN activation.

### 4.4 Northern blot

For DVGs of RNA viruses, Northern blot is a classic analytical method used to identify the size, emergence time, and retained key genomic fragments of DVGs (Table 1). This method separates molecules by size via electrophoresis. Radioactively or fluorescently labeled probes are then used to hybridize with specific DVG fragments, thereby enabling the detection and relative quantification of DVGs. Researchers initially detected naturally occurring DVGs in infectious bronchitis virus (IBV)-infected cells by means of northern blot analysis, which revealed that their genomes contained deletions of 1.1 kilobases (kb) at the 5′ end, 6.3 kb in the middle, and 1.6 kb at the 3′ end. The structural integrity of these DVGs was confirmed through RT-PCR and sequencing (Penzes et al., 1995). Additionally, northern blot analysis can be used to verify the selection and enrichment trends of DVGs during serial passage (Hernandez et al., 1996; Kang et al., 1985). A study analyzing DVGs of SARS-CoV-2-infected cells at different passage stages via northern blot employed a series of specific probes (A-G) for comprehensive coverage of the SARS-CoV-2 genome, identifying distinct dominant DVG populations in the early and late passage stages (Girgis et al., 2022). Furthermore, this method can assist in validating RNA sequencing results. For example, the 5 kb DVGs that were confirmed by northern blot exhibited consistent size and retained genomic fragments (such as the Nsp1-10 fusion sequence) with those resolved by direct RNA sequencing (Girgis et al., 2022).

## 5 The evolution of DVGs

After infecting the host, viruses must continually modify their genomic characteristics in order to respond to the environment. Meanwhile, host cells constantly adjust their status to resist viral invasion, leading to the coevolution of hosts and viruses (Knoll et al., 2024; Warger and Gaudieri, 2022). Among these processes, the evolution of DVGs has been confirmed to play a key role in viral environmental adaptation and enhanced survival capabilities (González Aparicio and López, 2024; Vignuzzi and López, 2019; Xu et al., 2017). The absence of key genes implicated in replication, packaging, and other vital processes necessitates the reliance of DVGs on helper viruses for the provision of proteins or critical enzymes (Huang and Baltimore, 1970; Hillung et al., 2024). This dependency directly restricts their transmission range and dictates that their evolutionary direction must be compatible with the genotype of the helper virus (Jaworski and Routh, 2017). Furthermore, DVG evolution is primarily influenced by factors such as competition for replication resources and host immune pressure (Manzoni and López, 2018; Mendes and Russell, 2021). Consequently, high-frequency genomic variations occur with great frequency, thus enabling the rapid selection of strains with stronger adaptability (Elena, 2023).

In the evolution of DVGs, the pressure exerted by long-term persistent viral infections provides the evolutionary driving force for the accumulation of replication errors and adaptation to the host environment in DVGs. On the one hand, as truncated viral genomes, DVGs undergo independent mutations via the template switching mechanism during replication selection during continuous replication. Conversely, persistent infections have been observed to induce specific deletions in DVGs to adapt to the long-term host cellular environment (Hillung et al., 2024). A study on the evolution of coronavirus DVGs demonstrated that DVGP50d-2 and DVGP90d-1 exhibited nucleotide deletions relative to the full-length genome during 50-day and 90-day persistent infections, respectively. This truncated structure may reduce viral consumption of host resources, thereby facilitating the persistence of DVGs in long-term infections (Lin, Tam et al., 2023). However, in the evolutionary process of SARS-CoV-2 DVGs, studies have found that with the continuous mutation of strains, the occurrence frequency of DVGs gradually decreases, and DVGs in key regions (such as near the S1/S2 cleavage site of the spike protein) have progressively disappeared. The retention of functional spike protein regions has been confirmed to enhance the fusion efficiency between viruses and host cells, thereby improving viral adaptability within the host (Campos et al., 2025; Kemp et al., 2021). Furthermore, innate immune pressure from the host is also a key driving force for DVG evolution. Following recognition by innate immune cells, DVGs must adjust their sequences or structures in order to evade immune attack. For example, following the activation of innate immunity with polyinosinic-polycytidylic acid (poly IC), DVGP100dIC-1 from coronaviruses did not acquire new mutations but avoided recognition by host immune mechanisms by maintaining a specific truncated structure (Lin, Tam et al., 2023). Antiviral drugs also play a significant role in the evolution of DVGs, promoting drug-resistant adaptive mutations in DVGs. After the administration of the antiviral drug remdesivir, the DVGs underwent continuous mutations to adjust replication-related sequences, while the diversity of DVGs decreased, with only the adaptive DVGs capable of replicating in the drug environment remaining (Lin, Chen et al., 2023). Indeed, DVG evolution shares the same core logic as viral evolution, both being the result of viral adaptation to the host environment and selective pressures.

### 5.1 The competition and cooperation between DVGs and full-length viral genomes

The evolution of DVGs is influenced by selective pressures and is closely associated with the competition and cooperation between DVGs and FLVGs. In addition to inhibiting the overall viral replication efficiency by interfering with FLVGs’ replication and restricting FLVGs spread through activating host immunity, the cooperative state between the two can also jointly maintain persistent viral infections. For instance, in shaping the immune microenvironment, the IFN response induced by DVGs has been shown to inhibit rapid viral replication, avoid prematurely killing host cells, and indirectly create a survival niche for FLVGs. Researchers administered DVGs into mice via intraperitoneal or intranasal injection and found that DVGs could protect mice from multiple RNA viruses, prevent lethal infections, and potentially contribute to long-term viral survival within the host (Xiao et al., 2021). Furthermore, the IFN response primarily acts on adjacent uninfected cells, inducing them to enter an antiviral state, which further protects infected cells and creates a stable microenvironment for viral replication and DVG accumulation. Consequently, in such circumstances, the long-term survival of viruses within the host becomes a possibility. The defective genomes of the hepatitis C virus (HCV) have been confirmed to facilitate persistent infections by upregulating HCV replication efficiency and enhancing viral particle release (Karamichali et al., 2018).

In the context of protracted infections, the interplay between DVGs and FLVGs is further exemplified by the observation that the persistent existence and accumulation of DVG diversity are contingent on FLVGs-mediated superinfection. The capacity of intact viruses to supply critical enzymes for DVGs is pivotal in ensuring their normal transcription and translocation to the cytoplasm. In a study related to HIV infection, researchers found that DVG RNA competes with FLVG RNA for packaging into viral particles, forming infectious particles carrying DVG RNA. After these particles infect new CD4^+^ T cells, DVG RNA reverse transcribes into the host genome and accumulates mutations continuously. Ultimately, an increasing number of DVGs with the same lethal deletions but distinct sequences emerge in FLVGs, thereby endowing DVGs with high diversity. This is a primary factor in the evolution of DVGs (Hariharan et al., 2025).

### 5.2 Evolutionary model of DVGs

In order to enhance comprehension of the evolution of DVGs, researchers have developed a range of mathematical and computational models. These models simulate the interactions between DVGs and FLVGs under different conditions, thereby enabling predictions of the dynamic changes of DVGs within viral populations. For instance, Sharov et al. (2023) constructed two mathematical models (ODE1 and ODE5) based on ordinary differential equations (ODEs) to elucidate the dynamic interactions between wild-type Zika virus (ZIKV) and naturally occurring DVGs (Sharov et al., 2023). The ODE5 model integrates intercellular infection dynamics with intracellular molecular dynamics. The intercellular level follows the transmission logic of “infection-viral release,” while the intracellular level incorporates additional variables such as wild-type genome replication, DVG genome replication, and viral protein synthesis. The integration of viral titer data and NGS haplotype data within the model resulted in the derivation of core parameters that fell within biologically established ranges, thereby substantiating high consistency with experimental data (Sharov et al., 2023). Additionally, certain computational methods have been employed to screen for highly adaptive DVGs. By identifying advantageous traits selected and retained during evolution, these methods allow reverse inference of the driving forces, conserved rules, and divergence directions of DVG evolution. Rezelj et al. developed a nested neighborhood algorithm that localizes deletion-enriched regions in a two-dimensional plane and tests their significance using the Poisson distribution. Subsequently, the relative adaptability of DVGs is evaluated via z-scores and followed by the validation of the conservation of open reading frame (ORF) retention, ultimately screening out highly adaptive DVGs (Rezelj et al., 2021). The development of these models not only helps explain the DVG-related phenomena observed experimentally, but also provides a theoretical basis for the development of DVG-based antiviral strategies.

## 6 Application of DVGs

DVGs are currently regarded as one of the effective strategies for combating viral infections, as they can interrupt normal viral replication processes and induce a certain level of innate immunity (López, 2014). To date, a variety of DVG-based antiviral agents have been developed. A number of studies have proposed the use of DVGs as alternatives to traditional vaccine adjuvants, due to their capacity to mimic viral infections and efficiently stimulate immunity (Linder et al., 2021; Fisher et al., 2022). Furthermore, DVGs have demonstrated considerable potential for application in cancer therapy, auxiliary vaccine production, and fundamental research (Wu et al., 2025).

### 6.1 Antiviral drugs

In the treatment of certain viral infections, DIPs/DVGs are regarded as a promising alternative research direction, owing to the absence of vaccines or antiviral agents with well-defined functions. Notably, the presence of DVGs can substantially augment host immune responses and impede viral replication. A plethora of studies on the antiviral effects of natural DVGs derived from paramyxoviruses, alphaviruses, flaviviruses, and coronaviruses have demonstrated their therapeutic potential in animal models (Brennan and Sun, 2024). A study was conducted in which DVGs with broad-spectrum antiviral activity from the Carib and LOL strains of Chikungunya virus (CHIKV) were screened. These DVGs were shown to circumvent the conventional constraints associated with targeting a singular virus. Specifically, they were found to curtail viral titers to levels that fell below the threshold of detection in closely related alphaviruses (e.g., Sindbis virus) (Levi et al., 2021). These results indicate that certain DVGs may target conserved replication machinery shared by the alphavirus genus, thereby conferring broad-spectrum activity against alphaviruses. Furthermore, naturally occurring DVGs of Nipah virus have also been shown to reduce viral titers by more than 1,000-fold. However, the antiviral mechanism of these DVGs solely stems from competing with FLVGs for replication resources, independent of the induction of interferon responses (Welch et al., 2020).

Therapeutic interfering particles (TIPs), which are artificially designed, can be optimized in their genomes to possess stronger replication competitiveness than wild-type DVGs (Fatehi et al., 2021; Welch et al., 2022; Pitchai et al., 2024). This enables them to sequester replication resources more efficiently and potently inhibit the replication of FLVGs (Lin et al., 2025). Xiao et al. created engineered therapeutic interfering particles (eTIPs) by deleting the capsid-coding region of poliovirus. Following administration, mice exhibited resistance to RNA viruses such as enteroviruses, influenza viruses, and SARS-CoV-2, with this resistance mediated by local and distal type I interferon responses (Xiao et al., 2021). Furthermore, researchers constructed DVGs from ZIKV that retain ORFs while deleting structures such as NS1. These highly adaptive DVGs reduced viral transmission rates by 90% in mosquito models and decreased viral loads in the bloodstream and target organs (e.g., brain, ovaries) in mouse models (Rezelj et al., 2021). However, research on DVGs as antiviral agents still has certain limitations. Most studies have focused only on the short-term safety of DVGs in mice, neglecting to address the long-term safety concerns arising from their persistence in organs (Dedieu et al., 1997). Furthermore, the precise mechanisms by which DVGs prevent viral infections remain to be elucidated, necessitating further optimization of research protocols *in vivo*.

### 6.2 Vaccine adjuvant

Antigenic components, including inactivated viruses and recombinant proteins, which constitute the fundamental elements of most vaccines, exhibit a low level of immunogenicity, characterized by a weak intensity and brief duration of the induced immune responses (Forsyth et al., 2019; Li et al., 2023). There is an urgent need to develop novel and effective vaccine adjuvants to enhance vaccine-induced immune responses, primarily by targeting innate immunity to drive adaptive immunity and extend the duration of vaccine protection (Sun and López, 2017). DVGs possess considerable potential as vaccine adjuvants (Yount et al., 2006; Coleman and Shukla, 2013). In viral infections, DVGs have been shown to be recognized by PRRs such as RIG-I and MDA5, and to promote the function of dendritic cells (DCs) (Sprokholt et al., 2017; Yang et al., 2019). For instance, oligonucleotides derived from RSV DVGs efficiently activate the RLR signaling pathway (Felt et al., 2021). This immunostimulatory activity is independent of intact viruses and not inhibited by immune escape proteins (e.g., NS1, NS2), enabling it to enhance the protective immune responses of vaccines. In fact, most current studies focus on developing adjuvants using DVGs derived from SeV, attributed to their unique interferon inducer function (Johnston, 1981). Researchers developed short-chain optimized DVG-derived molecules from SeV. The subcutaneous injection of these molecules into mice induced the expression of various proinflammatory cytokines, including IL-1β and TNF-*α*, and promoted the accumulation of DCs in the draining lymph nodes. Furthermore, the co-injection of these molecules with RSV resulted in higher anti-RSV antibody levels and a broader range of antibody subtypes (Mercado-López et al., 2013a). Similarly, SeV-derived DVG oligonucleotides, when employed in conjunction with IAV vaccines, exhibited significant type I immunity-oriented adjuvant functions, encompassing substantial promotion of IgG2c antibody production and activation of pivotal cellular immunity (Gnazzo et al., 2025). It is worth noting that, due to their dose-dependent nature and the cytokine storm induced by their continuous replication, the use of DVGs as adjuvants may necessitate the development of short DVGs comprising small functional units, or the selection of DVGs with low immunostimulatory activity (Mercado-López et al., 2013b). This would require alongside improvements to drug delivery systems to achieve targeted, sustained-release delivery. Furthermore, additional animal studies are required to establish the minimum effective dose and the safety threshold (Frensing et al., 2014).

### 6.3 Vector

DVGs, which lack partial viral genes, possess remaining genomic capacity that can be used to load foreign genes. Coupled with their characteristic of efficient replication in the presence of helper viruses, this directly enables high-efficiency expression of foreign genes to produce large quantities of therapeutic proteins or antigens (Roy et al., 2010). DVGs used as vectors are mainly concentrated in HSV, a feature attributable to HSV’s substantial vector capacity and its capacity to infect non-dividing cells (Lachmann, 2004). Previous studies suggested that vectors engineered to mimic DVG function by genetically modifying the HSV genome to eliminate its autonomous replication capacity have potential applications in gene therapy, cancer treatment, and neurological disease research (Burton et al., 2002; Le Hars et al., 2025). These studies knocked out essential immediate-early (IE) genes, such as ICP4 or ICP27, to prevent viral spread and subsequent infection. However, incomplete knockout results in apoptosis of a large number of non-neuronal cells, while complete knockout leads to epigenetic silencing of the viral genome and failure of transgene expression. To address this issue, Miyagawa et al. inserted a transposon expression cassette into the latency-associated transcript (LAT) locus while fully knocking out IE genes, or alternatively, they flanked the transgene with LAT elements at other genomic locations. This approach ensured sustained transcription of the transgene in non-neuronal cells (Miyagawa et al., 2015). The successful construction of HSV vectors has confirmed the feasibility of viral DVGs as gene vectors. Nevertheless, further exploration is required to develop methods for overcoming the heterogeneity of natural DVGs, laying the foundation for the development of next-generation gene therapy and vaccine technologies based on viral interference.

Although DVGs have potential as drugs and adjuvants due to their broad-spectrum antiviral and immune-activating capabilities, several obstacles must be overcome to achieve true clinical translation. With regard to the therapeutic window, Welch et al. noted that administration one day after viral challenge provides only minimal protection. It is only effective when administered prophylactically prior to exposure or concurrently with infection, and is not suitable for post-exposure treatment in humans (Welch et al., 2026). Furthermore, as the production of DVGs requires the use of live, infectious viruses. This necessitates reliance on high-level biosafety laboratories throughout the process and makes it difficult to eliminate live viruses from the mixture completely using traditional physical separation and UV inactivation methods. This poses a key obstacle to clinical translation (Welch et al., 2023). New preparation platforms, such as the BSL-2-compatible NiVΔF replicon (VRP) production platform using non-infectious NiV, can enhance the safety of the resulting DIPs. however, the titer is only 55 percent of that achieved by traditional methods, and further scaling up of production capacity and cost optimisation are required (Welch et al., 2026). It is worth noting that DIPs are currently classified as novel virus-like therapeutic particles and there are no existing regulatory guidelines for their approval. The resulting regulatory uncertainty has also slowed the pace of their clinical translation.

## 7 Conclusion

With the advancement of viral isolation and purification technologies, research on the functions and mechanisms of action of DVGs has regained attention. As incomplete genomic variants generated during viral replication, DVGs compete with FLVGs for replication resources and trigger innate immunity. This endows them with greater prominent effectiveness and universality than conventional methods in preventing viral pathogenicity. However, there are still many research gaps and significant challenges in this field that require be urgently addressed.

Firstly, research into the application of DVGs remains at the preclinical stage. Currently, there is a lack of reliable data on their actual efficacy in humans, and their true efficacy, safety, and dose–response relationships in the human body remain unclear (Li et al., 2026). In addition, the functional stability of DVGs in different viral infection microenvironments is uncertain, and the differences in their mechanisms of action across host and viral systems have yet to be elucidated, which severely limits the clinical translation and widespread application of DVGs (Yan et al., 2025). To address these issues, future research must focus on advancing the precise construction and optimisation of engineered DVGs. Biotechnological approaches such as gene editing and sequence modification, should be precisely regulate the structural characteristics, replication efficiency and immune-activating capacity of DVGs to enhance their targeting ability and stability. Furthermore, particular attention should be paid to emerging therapeutic strategies involving DVGs that combine cutting-edge methods, such as multi-omics and artificial intelligence-based screening, to explore various treatment regimens, including both monotherapy and combination therapy with DVGs.

In summary, DVGs offer a entirely new perspective for virological research and clinical applications. As relevant technologies continue to mature and research frameworks are further refined, the targeted modification of engineered DVGs and iterative innovations in novel antiviral treatment strategies will accelerate the transition of DVGs from basic research to clinical application. DVGs are expected to become a core technological tool in the fight against global viral infectious diseases and in overcoming challenges in public health prevention and control.
